# Magnetic Biochar Prepared with *Rosa roxburghii* Residue as Adsorbents for Congo Red Removal

**DOI:** 10.3390/ma18061306

**Published:** 2025-03-16

**Authors:** Xiaojuan Zhang, Xueqin Yang, Feiran Xie, Xianglan Chen, Yutao Zhang, Qiuyun Zhang

**Affiliations:** 1 School of Chemistry and Chemical Engineering, Anshun University, Anshun 561000, China; zxj9583@126.com (X.Z.); 18722907071@163.com (X.Y.); xie3533289541@126.com (F.X.); Chen_xianglan2025@163.com (X.C.); 2 Key Laboratory of Agricultural Resources and Resources and Environment in High Education Institute of Guizhou Province, Anshun 561000, China

**Keywords:** *Rosa roxburghii* residue, magnetic biochar, Congo red, adsorption

## Abstract

In this work, magnetic biochars (MBCs) were produced with the chemical coprecipitation method. The resulting materials were dried at 50 °C for 12 h and characterized via SEM-EDS, XRD, FT-IR, BET, TGA, and VSM techniques to evaluate their efficacy in removing Congo red (CR). The effects of solution pH, CR concentration, MBC1:1 mass, and a variety of ions on the adsorption performance were systematically examined. According to the experimental results, for 200 mL of 50 mg/L CR, the highest adsorption capacity of 20 mg MBC1:1 was 172.88 mg/g in a 2 h period at pH 7. Additionally, the pseudo-second-order (PSO) model-based kinetic analysis exhibited that the process of adsorption adhered to this model. Furthermore, the interaction between MBC1:1 and CR was best described by Langmuir multilayer adsorption, according to isotherm analysis. All of these theoretical and practical findings point to the great potential of MBC1:1 as adsorbents for the applications of wastewater treatment.

## 1. Introduction

Due to the growth of urbanization and industry, a considerable amount of dye effluents is being released into aquatic environments on a regular basis [1,2]. During the dyeing procedures, around 20% of the dyes are lost and end up in the water [3]. The majority of dyes tend to collect in nature because they are durable against degradation by any biological or chemical process. Additionally, they are stable against oxidizing chemicals and sunlight; they disrupt the ecological equilibrium by blocking sunlight penetration. Among a variety of dye categories, Congo red (CR) is a typical anionic diazo dye that is frequently applied in laboratory experiments, textiles, along with other commercial products due to its water solubility and color stability [4], and it can cause lung cancer, gastrointestinal irritation, weakness, and anorexia [5].

There are now a number of biological, chemical, and physical treatments reported for dye remediation [6,7,8,9]. Adsorption technologies are thought to be among the most competitive of the suggested treatment approaches because of their possible low cost, high efficiency, ease of design, and ease of use [10]. Choosing the right adsorbent for water treatment is crucial to attaining an optimal pollutant-removing performance [11]. In the last several years, many attempts have been conducted for the development of novel adsorption materials with high removal efficiency and low cost. Among the many adsorption materials available, biochar (BC) has become a widely applied adsorption material [12]. Its abundant porous structure gives it a considerably specific surface area together with a large number of attachment sites for pollutant adsorption. BC can also be made from a variety of forestry and agricultural wastes produced by human activity as raw materials [13]. BC has been produced successfully with various feedstocks, encompassing pineapple [14], banana pseudo-stem [15], Cymbopogon citratus [16], Rhizophora mucronate [17], cassava root-husk [18], etc. However, further procedures like filtration and centrifugation are needed to separate the powdered BC from ambient media. Secondary contamination may also arise from the processing of BC [19]. Magnetic nanoparticles loaded onto the BC surface have been employed in earlier research. For instance, Serife et al. [20] produced MBC loaded with Fe_3_O_4_ using acorn cups, and they were able to achieve a largest adsorption capacity of 52.63 mg/g for MB. Yan et al. [21] created magnetic particle-loaded BC utilizing a straightforward secondary grinding loading approach, and realized an adsorption capacity of 85 mg/L for MG. A BC-CoFe_2_O_4_-CS magnetic composite made from waste cardboard was studied by Cheng et al. [22] as an effective adsorbent, reaching a maximal adsorption capacity of 500.6 mg/g for methyl orange. Zhang et al. [23] produced magnetic self-nitrogen-doped BC utilizing K_3_[Fe(C_2_O_4_)_3_] as an iron supplier and activator, reaching outstanding adsorption capacities of 320 mg/g for Rhodamine B along with 1360 mg/g for CR.

*Rosa roxburghii* residue, a byproduct generated during the processing of *Rosa roxburghii*—a distinctive agricultural product from Anshun, Guizhou Province, China—is abundant in cellulose. *Rosa roxburghii* residue is primarily utilized in the production of fruit vinegar and as feedstock for animal feed. In prior research, our team successfully prepared biochars from *Rosa roxburghii* residue and discovered their significant adsorption capacity for methylene blue and rhodamine B in aqueous solutions. However, during the adsorption process of organic dyes, the biochars from *Rosa roxburghii* residue exhibited challenges including difficulty in recovery and suboptimal adsorption efficiency for CR. In this study, the biochar and three kinds of magnetic biochars (MBC1:1, MBC1:2, and MBC2:1) were prepared using *Rosa roxburghii* residue as a raw material by chemical coprecipitation. The adsorption performance of the biochar and three kinds of magnetic biochars is investigated with Congo red (CR) as the target dye. Investigations were conducted into the influences of CR concentration, adsorbent dosage, pH, and a variety of ionics on the CR adsorption by MBC1:1. Isotherm and kinetic models were exploited to study the adsorption mechanism, as well as the molecular diffusion process, separately. The recyclability of MBC1:1 was investigated concurrently. MBC performs better in adsorbing dyes owing to its greater specific surface area together with its micropore structure.

## 2. Materials and Methods

### 2.1. Reagents

Hydrochloric acid (HCl), Ferrous sulfate heptahydrate (FeSO_4_·7H_2_O), Ferrous chloride hexahydrate (FeCl_3_·6H_2_O), Congo red (CR), anhydrous ethanol (C_2_H_5_OH), as well as Sodium hydroxide (NaOH), were provided by Sigma-Aldrich. Since all of the reagents employed were analytical grade, no additional purification was required. Additionally, all of the treatment procedures employed deionized water. Residue from the juice of *Rosa roxburghii* was acquired from the Anshun Food Processing Factory, Anshun City, Guizhou Province, China.

### 2.2. Magnetic Biochar Production

Preprocessing: After being rinsed with DI water, the *Rosa roxburghii* residue was dried at 50 °C. It was dried, then milled and sifted through a 100-mesh sieve to extract the powdered *Rosa roxburghii* residue, which was reserved for further use.

Carbonization: The powdered residue of *Rosa roxburghii* was densely packed into the crucible and encapsulated with tin foil to ensure air isolation, followed by pyrolysis at 500 °C for 1 h in a muffle furnace. The eventual product was referred to as the biochar (BC) after it cooled to room temperature.

**Figure 1:**
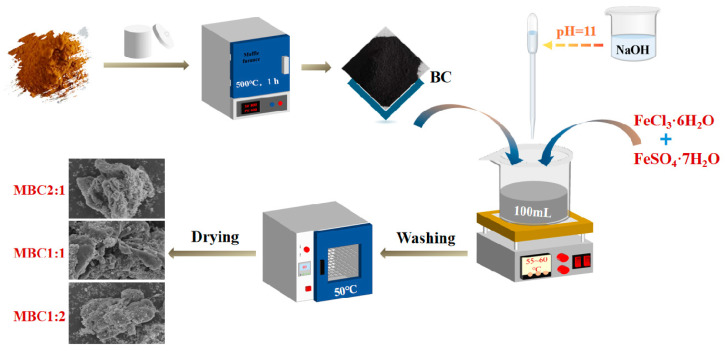
Flowchart of magnetic biochar production.

Magnetic Modification: Magnetic biochars (MBCs) were synthesized via chemical coprecipitation [24]. Specifically, 100 mL of DI water was applied to dissolve FeCl_3_·6H_2_O and FeSO_4_·7H_2_O at molar ratios of Fe^2+^:Fe^3+^ (1:2, 1:1, and 2:1). The solution was stirred magnetically until complete dissolution. Biochar (BC) was then added according to a mass iron-to-biochar ratio at 1:1, followed by heating the mixture to 60 °C under continuous stirring. To adjust the solution pH to 11.0, NaOH was added. The product was cleaned utilizing anhydrous ethanol and deionized water after standing for half an hour, and it was then dried for 12 h at 50 °C. The resulting magnetic biochars (MBCs) were designated as MBC1:2, MBC1:1, and MBC2:1, respectively.The detailed preparation process is shown in Figure 1.

### 2.3. Characterization of Biochars

Fourier transform infrared spectroscopy (FT-IR) was conducted using a PerkinElmer Spectrum 100 instrument, employing KBr pellets in the wavenumber range of 400–4000 cm^−1^ to identify chemical bonds and functional groups. In order to determine the crystallinity of the BC and MBC, the powder X-ray diffraction (XRD) patterns were documented on a Bruker D8 ADVANCE (Bruker, Berlin, Germany) with a Cu-Kα radiation source (λ = 0.15406 nm). The measurements were conducted over an angular range of 10° to 80° at a scanning rate of 5 °/min. The morphologies and elemental distribution of BC and MBC were observed utilizing field-emission scanning electron microscopy (SEM, Hitachi Regulus 8100, Tokyo, Japan) at 15 kV equipped with an energy-dispersive X-ray spectroscope (EDS) mapping analyzer. Automatic specific surface area and void analyzer (Micromeritics ASAP 2460 3.01, Micromeritics, Norcross, GA, USA) instrument was applied to record the N_2_ adsorption–desorption isotherms, and the pore size, along with the specific surface area, were identified in accordance with Brunauer–Emmett–Teller (BET) with the Micrometrics system. Thermogravimetric analysis (TGA) was implemented on an NETZSCH TG209F1 thermal analyzer under an N_2_ atmosphere with a heating rate of 10 °C/min. By using the multifunctional vibrating sample magnetometer (VSM, LakeShore 7404, Lake Shore Cryotronics, Inc. Westerville, OH, USA), the magnetic parameters of MBC1:1 were measured at room temperature with a maximum magnetic field intensity of 2T.

### 2.4. Adsorption Experiments

CR stock solutions with concentrations from 30 to 200 mg/L were produced. To conduct batch adsorption tests, 20–140 mg of biochars were added to 200 mL of CR dye solution at 50 mg/L. The mixture was gently stirred under stirring conditions at ambient temperature. In order to examine the influence of pH on CR adsorption, the dye solution pH was adjusted with NaOH (0.01 N) and HCl (0.01 N) solutions. The concentration of CR dye in samples that were taken at regular intervals of every 10 min were measured using an ultraviolet spectrometer (UV-5500PC, Shanghai Xiwen Biotech. Co., Ltd., Shanghai, China) at the peak absorption wavelength of 498 nm. *q_t_* and *R* were calculated using the following equations:

(1) $$q_{t}=\frac{\left(c_{0}-c_{e}\right)v}{m}$$

(2) $$R=\frac{\left(c_{0}-c_{e}\right)\times 100\%}{c_{0}}$$

Here, *q_t_* represents the biochar adsorption capacity for CR, mg/g; *R* denotes the removal rate of CR by biochars, %; *c*_0_ denotes the CR initial concentration, mg/L; *c_e_* is the equilibrium concentration of CR, mg/L; *v* is the volume of solution, L; and *m* represents the BC mass, g.

## 3. Results and Discussion

### 3.1. Biochar Characterization

#### 3.1.1. FTIR and XRD

**Figure 2:**
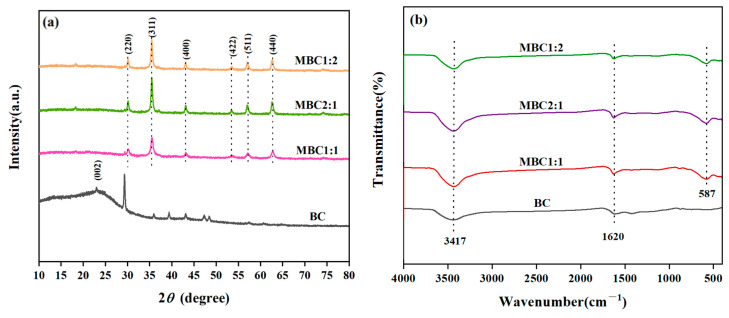
(**a**) XRD and (**b**) FT-IR spectrum of BC, MBC1:1, MBC2:1, and MBC1:2.

The XRD and FTIR patterns of different biochars are presented in Figure 2. As shown in Figure 2a, BC displays a broad diffraction peak centered at a value of 2θ at 23.2°, corresponding to the crystal plane (002), indicative of its irregular structure, namely the amorphous structure of amorphous carbon [25]. The samples MBC1:1, MBC2:1, and MBC1:2 all display relatively similar diffraction peaks, with the primary characteristic peaks at 62.6°, 57.0°, 53.4°, 43.1°, 35.5°, and 30.1°, corresponding to the crystal planes of Fe_3_O_4_ (440), (511), (422), (400), (311), and (220), separately [26], which suggests that Fe_3_O_4_ has been successfully loaded onto the BC. Furthermore, the broad diffraction peaks characteristic of BC were not observed on the surface of the MBC samples with varying proportions, indicating that Fe_3_O_4_ is highly dispersed on the biochar surface and exhibits significant magnetic properties.

Figure 2b exhibits that at 3417 cm^−1^, the broad peak was associated with the -OH stretching vibration of the water molecules absorbed by the material [27,28], and at 1620 cm^−1^, the characteristic peak correlated with the C=C and C=O bond in the biochars is evident [29]. Compared with the BC, the characteristic absorption peak of 587 cm^−1^ attributed to the Fe-O bending vibration was observed in MBC1:1, MBC2:1, and MBC1:2 [30], indicating successful synthesis of Fe_3_O_4_ on the biochar surface, which is in line with the findings of the XRD analysis.

#### 3.1.2. SEM-EDS

**Figure 3:**
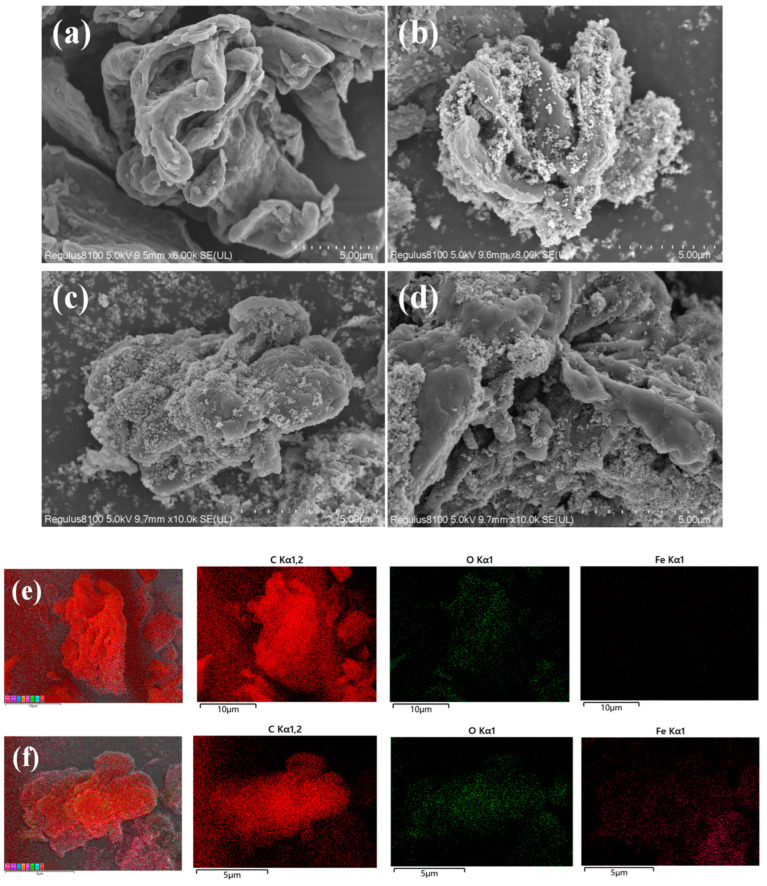
SEM images of the (**a**) BC, (**b**) MBC1:2, (**c**) MBC2:1, and (**d**) MBC1:1; EDS mapping of the BC (**e**) and MBC (**f**).

The elemental distribution and surface morphology of the biochar (BC) and magnetic biochars (MBC1:1, MBC2:1, and MBC1:2) were observed via a SEM and EDS detector (Figure 3). Figure 3a presents that the BC has a relatively smooth surface with few pores, indicating a relatively low specific surface area, which is not favorable for organic pollutant adsorption [31]. Figure 3b–d exhibits that after magnetic modification, there are more defects on the surface of the biochars, showing a concave–convex micro-polyhedron, which is conducive to increasing the specific surface area of the magnetic biochars, thereby enhancing their adsorption performance. In addition, it can be found that Fe_3_O_4_ nanoparticles adhere uniformly to the surface of magnetic biochars, which is mainly due to the nucleation and crystal growth of Fe_3_O_4_ [32]. These Fe_3_O_4_ crystals are closely arranged, and micro-agglomerations can be observed on the surface and gap of the magnetic biochars [33], which is attributed to the fact that, owing to magnetic dipole–dipole interactions, magnetic nanoparticles are typically prone to aggregate [34]. Furthermore, the EDS spectra of the BC (Figure 3e) revealed that the entire surface of the BC contained O and C elements, and the spectra of the MBC (Figure 3f) also indicated that Fe, O, and C elements were uniformly distributed on the MBC composite surface. In addition, EDS (Figure S1) revealed that, compared with the BC, the content of C in the MBC decreases while that of Fe and O increases, which indicates that during the formation of the MBC, the Fe–O bonds form. Both the FTIR and XRD spectroscopic analyses confirmed the presence of Fe–O functional groups and Fe elements, indicating that Fe_3_O_4_ was successfully loaded onto the surface of the biochars.

#### 3.1.3. BET

**Figure 4:**
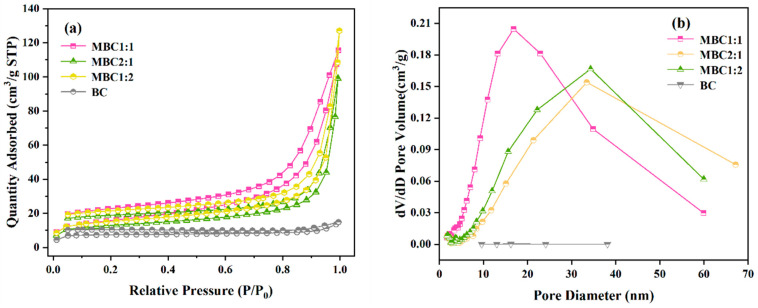
(**a**) N_2_ adsorption-desorption isotherms and (**b**) pore size distributions of BC, MBC1:1, MBC2:1, and MBC1:2.

The nitrogen adsorption–desorption isotherms and pore size distribution of biochars were depicted in Figure 4. The findings (Figure 4) exhibited the isotherms of the BC are classified as Type II, indicating a predominance of macropores with relatively fewer micropores present. The isotherms of the MBC are all of type IV, and they have obvious lag loops, which indicates that the MBC has microporous and mesoporous/large-pore structures [35,36]. Based on Table 1, the proportion of micropores in the BC is relatively low, while that of macropores is significantly higher. This porous structure is conducive to the loading of Fe_3_O_4_ particles and provides a large number of active sites. The total pore volume of the MBC is substantially greater than the micropore volume, indicating the presence of mesoporous and macroporous structures. This hierarchical porous architecture endows the material with numerous exposed adsorption sites and minimal diffusion resistance, which facilitates efficient adsorption of CR. Additionally, the specific surface areas for MBC1:2, MBC2:1, MBC1,1:1, and BC were 55.7369, 46.9072, 56.3398, and 30.8006 m^2^/g, respectively, with pore sizes of 14.1040, 13.0753, 12.7016, and 2.9758 nm. It can be inferred that magnetic modification can bring about higher specific surface areas and pore sizes, which may be related to the generation of more porous structures on the biochar matrix surface due to the aggregation of Fe_3_O_4_ nanoparticles [37]. The higher BET surface area may result from the MBC, which can lead to exposure of more active sites and increased catalytic activity. Among these samples, MBC1:1 has the largest specific surface area, promoting uniform mesopore distribution and providing numerous active sites, thereby enhancing Congo red (CR) adsorption [38].

#### 3.1.4. VSM

**Figure 5:**
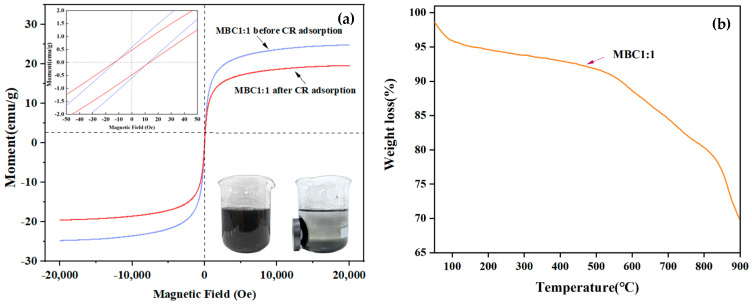
(**a**) VSM curves of MBC1:1 before and after CR adsorption; (**b**) TGA curve of MBC1:1.

Figure 5a shows the magnetic hysteresis loop before and after the MBC1:1 adsorption of CR. Before CR adsorption, the hysteresis loop of MBC1:1 is close to the “S” type. The saturation magnetization of MBC1:1 is 24.5 emu/g, the coercivity is 11.4Oe, and the remanence is 0.53 emu/g, which indicates that MBC1:1 has super paramagnetism. After the adsorption of CR, the saturation magnetization of MBC1:1 decreased to 19.4 emu/g, which may be related to the decrease of the mass fraction of magnetic particles after the material surface was occupied by CR molecules. Although it has decreased, MBC1:1 after adsorption of CR still has highly paramagnetic properties, which can ensure that MBC1:1 can effectively separate from the liquid in recycling and achieve good reuse value. It can also be observed that after MBC1:1 is recovered, the water becomes clearer from turbidity. The results of the SEM, EDS, FTIR, and XRD analyses confirm that Fe_3_O_4_ was successfully loaded onto the BC. The adsorbent with the relative high magnetism could be separated quickly and easily, avoiding discard into the environment.

#### 3.1.5. TGA

The TGA analysis of MBC1:1 is shown in Figure 5b. The MBC1:1 remained pleasantly stable up to 500 °C, demonstrating the comparatively high thermal stability of the adsorbent. A little mass loss was noted before 100 °C, and this was attributed to the water evaporating from the adsorbent [39]. Additionally, the dehydroxylation of MBC1:1 caused a considerable loss of weight between 100 °C and 550 °C. Interestingly, the breakdown of organic components, particularly cellulose, caused a dramatic weight loss peak over 550 °C [40]. Most importantly, MBC1:1 seems to have good stability, which is conducive to its recycling in the process of adsorbing CR because the breakdown temperature (550 °C) of the adsorbent is still marginally greater than the reaction temperature (500 °C).

### 3.2. Adsorption Experiment

The properties of the biochars and their efficacy in removing the target pollutant CR were examined in this work. For a comprehensive investigation, the adsorption period, adsorbent quantity, CR solution concentration, and pH were all methodically changed.

#### 3.2.1. Effects of Different Biochars

**Figure 6:**
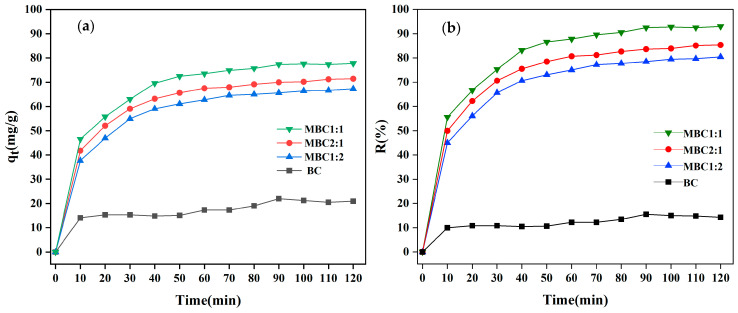
Results of (**a**) adsorption capacity and (**b**) removal rate for CR by BC, MBC1:1, MBC1:2, and MBC2:1.

The adsorption properties of the biochar (BC) and three types of magnetic biochars (MBC1:1, MBC2:1, and MBC1:2) towards CR were examined. Figure 6 exhibits that the adsorption effect of the three types of magnetic biochars on CR was significantly better than that of the biochar (BC), indicating that magnetic modification enhanced the adsorption capacity and removal rate of the BC for CR. Meanwhile, the adsorption curves of the BC and the three types of magnetic biochars towards CR presented three stages: rapid, slow, and equilibrium (Figure 6a,b). The adsorption rate was relatively fast in the early stage of adsorption, as the surface of the biochars possesses a high density of adsorption sites, allowing CR to be adsorbed rapidly. Subsequently, the adsorption rate slowed down, possibly owing to the fact that the number of available adsorption sites was decreased, and the occupied sites might repel CR in the solution, thereby affecting the adsorption rate [41]. As illustrated in Figure 6a,b, after 90 min, the adsorption capacity was basically stable. The adsorption capacities and removal rates of the BC, MBC1:1, MBC2:1, and MBC1:2 were 20.97 mg/g and 14.30%, 77.78 mg/g and 92.98%, 71.41 mg/g and 85.36%, and 67.29 mg/g and 80.44%, respectively. In line with the findings of the BET study, it was evident that MBC1:1 had the optimum adsorption impact on CR. The adsorption mechanism and performance of the magnetic biochars towards CR were investigated in the text that follows utilizing MBC1:1 as the adsorbent.

#### 3.2.2. Influence of Adsorbent Dosage

**Figure 7:**
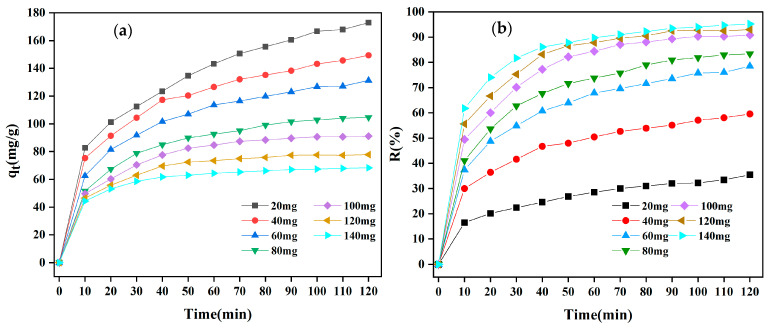
Impact of (**a**) adsorption capacity and (**b**) removal rate for MBC1:1 dosage on CR adsorption.

The adsorbent dosage is one of the factors that significantly affects the adsorption capacity; it provides the binding sites and a sizable surface area needed for dye adsorption on the adsorbent. The impact of adsorbent dosage on CR adsorption was investigated in 200 mL 50 mg/L CR dye solutions at dosages from 20 to 140 mg (Figure 7). The equilibrium adsorption capacity of MBC1:1 steadily declined as the dosage of MBC1:1 rose, although the CR removal rate increased. The largest adsorption capacity of MBC1:1 for CR dyes was 172.88 mg/g (Figure 7a), and the maximum removal rate was 35.42% (Figure 7b), when the adsorbent dosage was 20 mg. The adsorption capacity of CR dye dropped to 68.26 mg/g (Figure 7a) as the dosage was raised to 140 mg, while the removal rates rose to 95.2% (Figure 7b). The points of view of both the dye and the adsorbent might be employed to explain this event. First, the CR dye may be more completely adsorbed into the adsorbent surface as the MBC1:1 dose rises. The concentration of residual dye then drops, raising the removal rate of the dye [42]. Second, a substantial equilibrium adsorption capacity was achieved by the adsorbent when the dosage was minimal because the adsorption sites on its surface were primarily occupied [43,44]. Nevertheless, there is a reduction in the equilibrium adsorption capacity of the adsorbent with rising doses due to the fact that more adsorption sites are retained on the absorbent surface as the number of adsorption sites grows [45]. As a result, MBC1:1 might reach a higher equilibrium adsorption capacity when the concentration of dye is high enough.

#### 3.2.3. Impact of CR Initial Concentration

**Figure 8:**
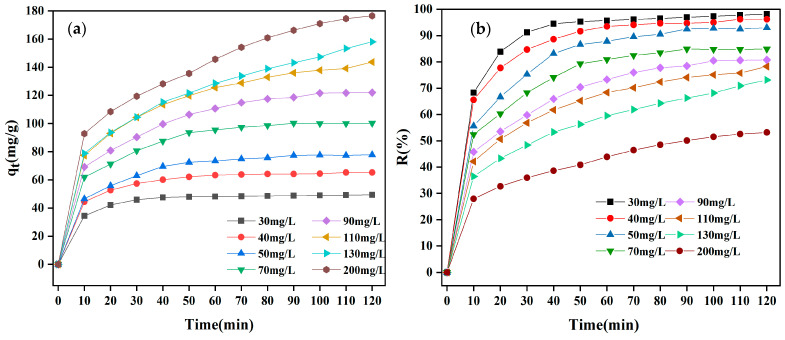
Impact of (**a**) adsorption capacity and (**b**) removal rate for the concentration of CR on adsorption of CR by MBC1:1.

The adsorption process of the CR solution was examined under the conditions of PH (7) and adsorbent dosage (120 mg/200 mL) (Figure 8). Figure 8 reveals that as the initial CR concentration was elevated from 30 to 200 mg/L, the adsorption equilibrium removal rate of CR by MBC1:1 decreased from 98.16% to 53.17% (Figure 8b). This reduction may be caused by the limited number of active sites on MBC1:1. At higher CR concentrations, CR initially adsorbs onto surface-active groups before diffusing into internal pores, leading to rapid occupation of the available adsorption sites and, consequently, reducing the CR removal efficiency [46]. Furthermore, the maximal adsorption capacity of MBC1:1 for CR was elevated from 49.39 mg/g to 176.55 mg/g (Figure 8a). This rise is likely due to the higher initial concentration providing a greater driving force for mass transfer, thereby raising the adsorption capacity of MBC1:1. With the progress of the adsorption, the adsorption sites on the adsorbent became saturated, causing a slow rise in adsorption until equilibrium was finally achieved.

#### 3.2.4. The Influence of pH Value

**Figure 9:**
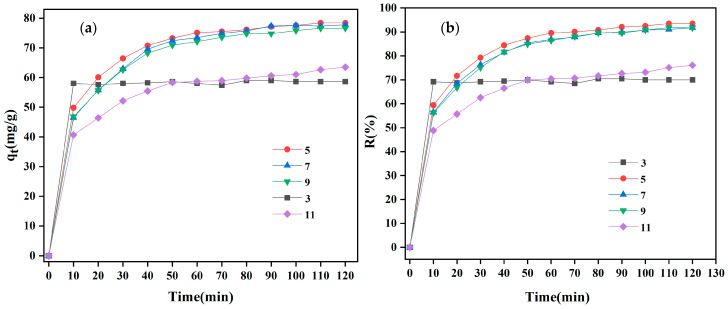
Effect of (**a**) adsorption capacity and (**b**) removal rate for pH value on adsorption of CR by MBC1:1.

In order to examine the influence of solution pH on the adsorption of CR, a 50 mg/L CR solution with pH values between 5 and 11 was incorporated into MBC1:1 adsorbent with a mass of 120 mg/200 mL. The results are presented in Figure 9. Figure 9 reveals that the MBC1:1 adsorption performance on CR remains relatively consistent within a pH range of 5 to 9, displaying the best adsorption efficiency with a removal rate of 93.49% (Figure 9b) and a maximal adsorption capacity of 77.78 mg/g (Figure 9a). However, the removal rate and adsorption capacity dropped to 76.07% and 69.94% (Figure 9b) at pH 3 and 11, corresponding to an adsorption capacity of 63.49 and 58.64 mg/g (Figure 9a), separately. At low pH levels, the carbonate and Fe_3_O_4_ components in MBC1:1 may react with H^+^ ions, leading to an increase in surface negative charge and, consequently, weakening the electrostatic attraction between MBC1:1 and the anionic dye CR [47]. Conversely, at high pH levels, the reduction in the surface positive charge of MBC1:1 weakens the electrostatic attraction between the negatively charged CR molecule and MBC1:1. Additionally, the existence of a large number of -OH ions will compete with CR molecules for the adsorption sites, considerably lowering the adsorption rate of CR by adsorbent materials [48]. These observations suggest that, in addition to electrostatic interactions, other forces (for instance, hydrogen bonding and π-π stacking) may also act as key players in the process of adsorption [49].

#### 3.2.5. Effect of Different Ionics

**Figure 10:**
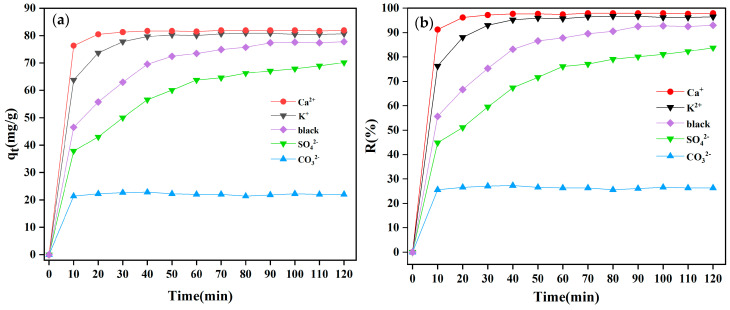
Effect of (**a**) adsorption capacity and (**b**) removal rate for different ionics on adsorption of CR by MBC1:1.

Real textile wastewater contained several inorganic ions in addition to dye molecules. The adsorption capability of the dyes was impacted by the competition of these inorganic ions for dye molecules with the MBC1:1 adsorption sites. Ca^2+^, K^+^, SO_4_^2−^, and CO_3_^2−^ were chosen for this investigation as analogues of other ions found in real water samples. The findings revealed in Figure 10a,b exhibited that with cations (K^+^ and Ca^2+^) added to the CR solution, the adsorption capacity and removal rate of MBC1:1 on anionic CR dye gradually increased. This is because K^+^ and Ca^2+^ are cations and can better neutralize anionic CR dye. Due to the more positive charge carried by Ca^2+^, the effect of Ca^2+^ is more significant, and the adsorption capacity rises from 77.78 mg/g to 81.90 mg/g. The MBC1:1 adsorption capacity for CR decreased from 77.78 to 70.17 mg/g by adding 0.1 mol/L SO_4_^2−^, which may be caused by the competition between anionic CR dye and SO_4_^2−^. The largest anion affecting the adsorption effect is CO_3_^2−^, and the adsorption rate decreases from 77.78 mg/g to 22.02 mg/g. The reason may be that in an acidic environment, CO_3_^2−^ will combine with a significant amount of H^+^ in the solution to form HCO_3_^−^, which reduces the H^+^ concentration and increases the solution pH.

### 3.3. Adsorption Kinetics

In this work, the CR adsorption kinetics by the magnetic biochar (MBC1:1) were accurately characterized using both the PFO and the PSO models. These models effectively display how the CR concentration varies over time during adsorption. Approximately 120 mg of MBC1:1 was added to 200 mL of CR dye solution at 30–200 mg/L. The mixture solutions were gently stirred under stirring conditions at ambient temperature. The concentration of CR dye in samples that were taken at regular intervals of every 10 min were measured using an ultraviolet spectrometer (UV-5500PC, Shanghai Xiwen Biotech. Co., Ltd., Shanghai, China) at the peak absorption wavelength of 498 nm. The fundamental process causing MBC1:1 adsorption of CR can be clarified by evaluating the degree of concordance between practice and theory via comparing experimental results with model predictions. The following dynamic equations are applied in the study of MBC adsorption kinetics for CR.

(3) $$\ln C_{t}/C_{0}=k_{1}t$$

(4) $$\frac{t}{q_{t}}=\frac{t}{q_{e}}+\frac{1}{k_{2}q_{e}^{2}}$$

In the above equations, *C*_0_ and *C_t_* represent the CR initial concentration and the concentration at time *t*, separately, *k*_1_ and *k*_2_ denote the rate constants for the kinetic reaction (L/min and g/mg·min), and *t* stands for the reaction time (min). *q_e_* and *q_t_* represent the concentration (mg/g) of the adsorbent at equilibrium and time *t*, separately.

**Figure 11:**
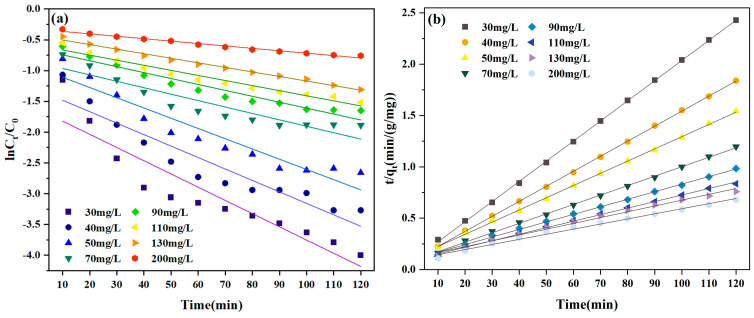
PFO kinetic (**a**) and PSO kinetic models (**b**) of CR by MBC1:1.

Figure 11 and Table 2 display the findings of the studies conducted by utilizing the PFO and PSO kinetic models. It is significant that the PSO kinetic model can adequately represent the CR adsorption employing the MBC1:1 adsorbent, since the R^2^ values produced from the PSO kinetic models perform better than those developed from the PFO kinetic models. The findings of the evaluation aligned with the experimental findings. Furthermore, the experimental and simulated data exhibit a high degree of consistency, indicating that the MBC1:1 adsorption CR was primarily chemisorption [50]. The surface abundance of active sites on the adsorbent and the intensity of the interaction between CR and the adsorbent are the main factors influencing the CR adsorption by the MBC1:1 adsorbent. By modeling the first- and second-order kinetics of the removal of CR by MBC1:1, the compatibility between practice and theory was examined.

### 3.4. Adsorption Equilibrium

The following equations and the Freundlich, along with the Langmuir, isotherm models were also applied to interpret the experimental data [51,52].

(5) $$\frac{C_{e}}{q_{e}}=\frac{C_{e}}{q_{m}}+\frac{1}{q_{m}b}$$

(6) $$\ln q_{e}=\frac{1}{n}\ln C_{e}+\ln k_{f}$$

**Figure 12:**
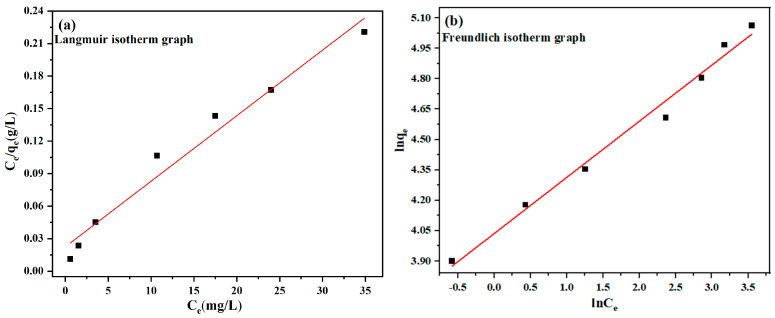
(**a**) Langmuir isotherm of CR onto MBC1:1; (**b**) Freundlich isotherm of CR onto MBC1:1.

Figure 12 and Table 3 display the characteristics of the Freundlich and Langmuir models utilizing 30–130 mg/L CR absorbed on 120 mg MBC1:1 while stirring for 120 min (Table S1). The determination coefficients (R^2^) for the Freundlich and Langmuir models were found to be 0.9771 and 0.9900, separately. The increased consistency presented by the Langmuir isotherm model indicates that the CR adsorption by MBC1:1 follows a multilayer adsorption pattern. Based on [53], the MBC1:1 adsorption method was found to be highly favorable for CR (n > 1), as indicated by the n values, which suggest that the adsorption process was favorable, at 2.77.

### 3.5. Adsorption Thermodynamics

The thermodynamics of CR removal were investigated to gain further insight into the adsorption mechanism of CR. MBC1:1 (120 mg) was incorporated into a CR solution at 50 mg/L and treated at various temperatures (T = 20 °C, 30 °C, and 40 °C) in comparison with ambient temperature. Thermodynamic properties (∆*G*^0^, ∆*H*^0^, and ∆*S*^0^) were assessed via the following equations:

(7) $$K_{0}=\frac{q_{e}}{c_{e}}$$

(8) $$\Delta G^{0}=-RT\ln K_{0}$$

(9) $$\ln K_{0}=\frac{\Delta S^{0}}{R}-\frac{\Delta H^{0}}{RT}$$

In this work, *R* and *K*_0_ denote the gas constant and Langmuir adsorption constant (L·mol^−1^), separately.

**Figure 13:**
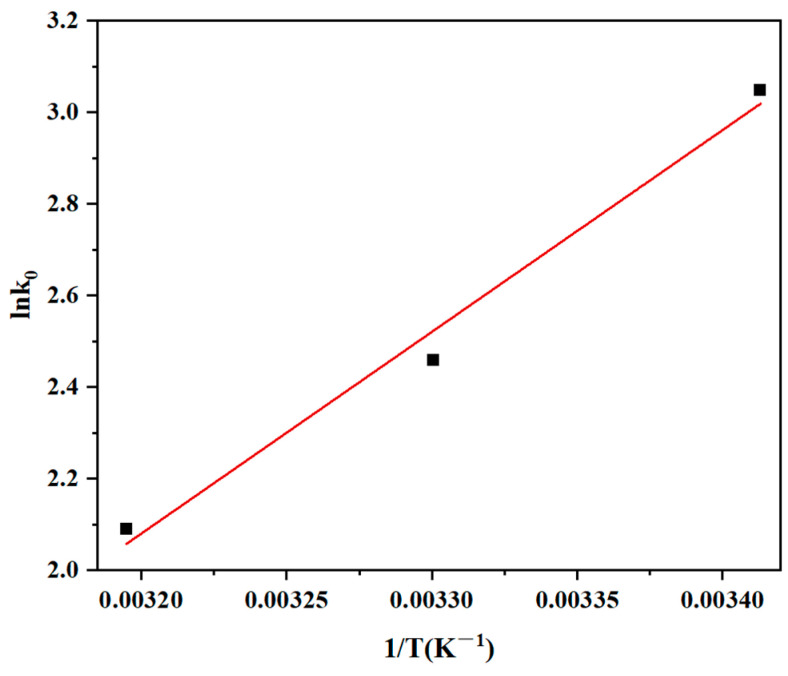
The thermodynamic equilibrium of CR adsorption by MBC1:1.

With a correlation value (R^2^) of 0.9745 (Figure 13), a substantial relationship between the logarithm of the Langmuir adsorption constant and the reciprocal of temperature (1/T) was found, highlighting the precision of these thermodynamic predictions. The findings (Table 4) demonstrated that the adsorption process was spontaneous as the ∆G^0^ of the CR adsorbed by MBC1:1 was negative during the 20–40 °C temperature range. The adsorption was exothermic, as evidenced by the negative ∆H^0^. Since the ∆S^0^ values are negative, it can be concluded that the process of adsorption reduced the randomness of the adsorbent at the adsorbate/adsorbent interface. To put it another way, it indicates that the dye molecules are freer and more random in the bulk of the solution than they are when they connect to the adsorbent surface [54].

### 3.6. Recyclability and Regeneration of MBC1:1 Adsorbent

Research on this material is more significant, as in practical adsorption applications, the more cycles of desorption and adsorption can be conducted, the lower the cost of the dye wastewater treatment. As a result, this study also examined the cycling properties of the adsorbent.

**Figure 14:**
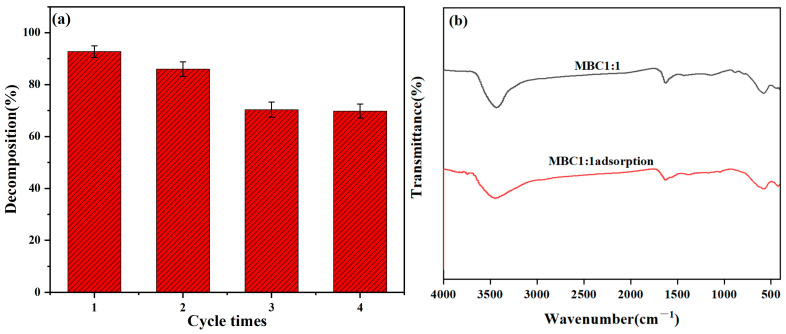
(**a**) Recyclability of MBC1:1 for CR adsorption; (**b**) FT-IR spectra of MBC1:1 prior to and after adsorption.

The recyclability of MBC1:1 was evaluated under the following conditions: 120 mg of adsorbent, 200 mL of 50 mg/L CR solution, and a 2 h adsorption time. For each cycle, the adsorbent was soaked in anhydrous ethanol and then separated by centrifugation. Figure 14a displays that the removal efficiency reduced from 92.73% to 69.81% after four consecutive adsorption cycles using MBC1:1. Additionally, the structural integrity of MBC1:1 after four cycles was examined using FT-IR analysis (Figure 14b). The FT-IR results indicate that the structure of MBC1:1 remains largely intact, demonstrating its good stability under the adsorption conditions. However, the observed decrease in removal efficiency during the fourth cycle may be attributed to incomplete regeneration of MBC1:1 or irreversible deactivation at some active sites [55].

### 3.7. Adsorption of CR Dye by Various Adsorbents

Table 5 reveals the results of adsorption by various adsorbents on CR. It is evident that in comparison to the MBC1:1 produced in this work, most adsorbents require higher adsorption dosages and longer adsorption times to complete adsorption, suggesting the magnetic biochar synthesized by the chemical coprecipitation method from *Rosa roxburghii* residue showed excellent adsorption properties. In addition, the modification or compounding of the magnetic biochar is very important to improve the adsorption performance of magnetic biochars. The outcome offers a method for creating magnetic biochars that have a high ability for adsorption utilizing *Rosa roxburghii* residue.

## 4. Conclusions

In this study, we successfully synthesized magnetic biochars (MBC1:1, MBC2:1, and MBC1:2) with superior adsorption properties. The structural characteristics of these materials were comprehensively analyzed with the advanced techniques, for instance, SEM-EDS, FTIR, XRD, VSM, and TGA. The effective removal of CR was achieved by utilizing these described magnetic biochars. The results indicate that magnetic modification significantly enhanced the adsorption capacity, with MBC1:1 exhibiting the best performance. Specifically, the maximal adsorption capacity of 172.88 mg/g was reached at a pH of 7, an adsorbent dosage of 20 mg of MBC1:1, and a concentration of 50 mg/L of CR. Furthermore, as the level of MBC1:1 raised to 120 mg and the concentration of CR lowered to 30 mg/L, an impressive removal efficiency of 98.18% was achieved. Experimental findings also revealed that cations (K^+^, Ca^2+^) promoted the adsorption of CR by MBC, whereas anions (CO_3_^2−^, SO_4_^2−^) inhibited it. Based on these experimental results, theoretical analysis suggests that chemisorption is a key component in efficient removal procedures by MBC1:1. This work highlights the efficient regeneration capabilities and excellent adsorption capacity of the adsorbent and offers a potential practical strategy for dye removal from water.

## Figures and Tables

**Table 1:** Data of biochars associated with pore structure and BET surface area.

Thermophysical Properties	BC	MBC1:1	MBC2:1	MBC1:2
BET surface area (m^2^/g)	30.8006	56.3398	46.9072	55.7369
Total pore volume (cm^3^/g)	0.01978	0.1564	0.1087	0.1280
Micropore volume (cm^3^/g)	0.007515	0.002823	0.008833	0.010611
Micropore area (m^2^/g)	21.1302	7.8049	21.5450	25.9734
Average pore size (nm)	2.9758	12.7016	13.0753	14.1040

**Table 2:** Kinetic parameters for the CR adsorption over the MBC1:1.

Mass (mg)	Concentration (mg/L)	PSO Model		PFO Model		
		k_2_ (g/(mg·min))	R^2^	k_1_ (L/min)	R^2^	q_e,exp_ (mg/g)
120	30	0.0196	0.9997	0.0215	0.8509	49.39
	40	0.0146	0.9998	0.0186	0.8883	65.23
	50	0.0119	0.9994	0.1663	0.8989	77.79
	70	0.0092	0.9989	0.0104	0.8448	100.20
	90	0.0074	0.9983	0.0096	0.9299	122.02
	110	0.0064	0.9976	0.0083	0.9569	143.63
	130	0.0057	0.9901	0.0074	0.9902	158.02
	200	0.0050	0.9898	0.0039	0.9803	176.55

**Table 3:** Summary of Freundlich and Langmuir isotherm constants for the CR removal by MBC1:1.

	**Langmuir Isotherm**		**Freundlich Isotherm**	
Adsorbent	1/*q_m_*	R^2^	1/n	R^2^
MBC1:1	0.0060	0.9665	0.2770	0.9845

**Table 4:** CR thermodynamic parameters obtained from the Langmuir model.

Van’t Hoff Equation	Temperature (°C)	∆G° (kJ/mol)	∆H° (kJ/mol)	∆S° (J/(mol·K))
Y = 4408x − 12.0	20	−7.4	−36.6	−99.8
	30	−6.2		
	40	−5.4		

**Table 5:** Adsorption of CR dye by various adsorbents.

Adsorbent	Adsorbent Dosage	Dye Concentration	Adsorption Time	Adsorption Capacity	Ref.
Fe_x_Co_3−x_O_4_ nanoparticle	0.05 g/500 mL	20 mg/L	240 min	128.6 mg/g	[56]
Activated carbon	0.8 g/L	200 mg/L	180 min	234 mg/g	[10]
(Chit/AILP-Kao) nanocomposite	150 mg/20 mL	20 mg/L	240 min	104.7 mg/g	[57]
Fe-BC	40 mg/20 mL	100 mg/L	90 min	45.96 mg/g	[58]
Magnetic self-nitrogen-doped biochar	20 mg/200 mL	1000 mg/L	500 min	1360 mg/g	[23]
magnetic biochar (MBC1:1)	20 mg/200 mL	50 mg/L	120 min	172.88 mg/g	This study

## Data Availability

The data implemented to support the results of the study are included within the manuscript.

## Supplementary Materials

The following supporting information can be downloaded at: https://www.mdpi.com/article/10.3390/ma18061306/s1, Figure S1: EDS image of BC, MBC1:1, MBC2:1, and MBC1:2; Figure S2: The mechanism of magnetic formation; Table S1: Experimental data at equilibrium; Table S2: Experimental data of Recyclability and regeneration of MBC1:1 adsorbent.
