# Supplementary material for: Magnetic Biochar Prepared with Rosa roxburghii Residue as Adsorbents for Congo Red Removal
Source: Materials (Basel). 2025 Mar 16;18(6):1306. doi: 10.3390/ma18061306 (PMC11943761; doi:10.3390/ma18061306)
Supplement: Supplementary file 1 [file materials-18-01306-s001.zip › materials-3501986-supplementary.pdf]

Supporting Information (SI) for:

Magnetic Biochar Prepared with *Rosa roxburghii* residue as Adsorbents for Congo red Removal

Xiaojuan Zhang [1,2], Xueqin Yang[1], Feiran Xie[1], Xianglan Chen[1], Yutao Zhang [1,2*], and Qiuyun Zhang[1,2*]

1 School of Chemistry and Chemical Engineering, Anshun University, Anshun, Guizhou, China; zxj9583@126.com (X.Z.); 18722907071@163.com (X.Y.); xie3533289541@126.com (F.X.); Chen_xianglan2025@163.com (X.C.)

2 Key Laboratory of Agricultural Resources and Resouces and Environment in High Education Institute of Guizhou Province, Anshun, 561000, China

* Correspondence: zyt0516@126.com (Y.Z.); sci_qyzhang@126.com (Q.Z.)

Text S1. Biochars characterization

Fourier transform infrared spectroscopy (FT-IR) was conducted using a PerkinElmer Spectrum 100 instrument, employing KBr pellets in the wavenumber range of 400-4000 cm-1 to identify chemical bonds and functional groups. In order to determine the crystallinity of the BC and MBC, the powder X-ray diffraction (XRD) patterns were documented on a Bruker D8 ADVANCE (Bruker, Berlin, Germany) with a Cu-Kα radiation source (λ=0.15406 nm). The measurements were conducted over an angular range of 10° to 80° at a scanning rate of 5 °/min. The morphologies and elemental distribution of BC and MBC were observed utilizing a field-emission scanning electron microscopy (SEM, Hitachi Regulus 8100, Tokyo, Japan) at 15kV equipped with an energy-dispersive X-ray spectroscope (EDS) mapping analyzer. A small amount of sample was carefully placed on the conductive adhesive. Subsequently, gold sputtering was performed using a Quorum SC7620 sputter coater for 45 seconds at a current of 10 mA. Morphological and energy-dispersive X-ray spectroscopy (EDS) mapping analyses were then conducted. For morphological imaging, an accelerating voltage of 3 kV was applied, while for EDS mapping, an accelerating voltage of 20 kV was used. The SE2 secondary electron detector was employed for these measurements. Micromeritics ASAP 2460 3.01 (USA) instrument was applied to record the N2 adsorption-desorption isotherms, and the pore size along with specific surface area were identified in accordance with Brunauer-Emmett-Teller (BET) with Micrometrics system. Thermogravimetric analysis (TGA) was implemented on a NETZSCH TG209F1 thermal analyzer under a N2 atmosphere with a heating rate of 10°C/min. By using the multifunctional vibrating sample magnetometer (VSM, LakeShore 7404, USA), the magnetic parameters of MBC1:1 were measured at room temperature with a maximum magnetic field intensity of 2T.

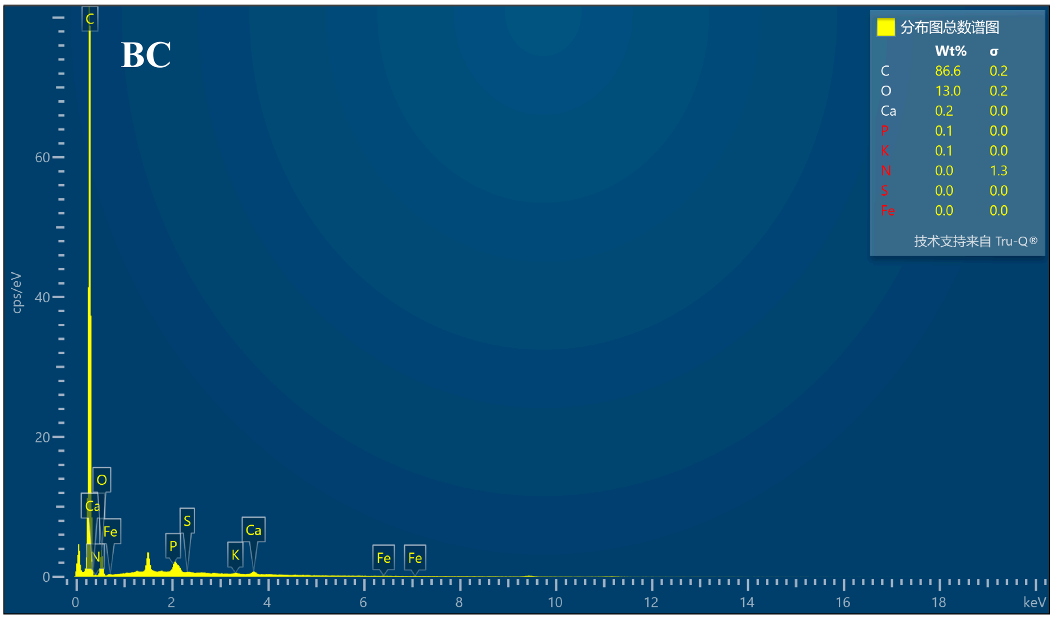
BC
分布图总数谱图
Wt% σ
C 86.6 0.2
O 13.0 0.2
Ca 0.2 0.0
P 0.1 0.0
K 0.1 0.0
N 0.0 1.3
S 0.0 0.0
Fe 0.0 0.0
技术支持来自 Tru-Q®
cps/eV
keV

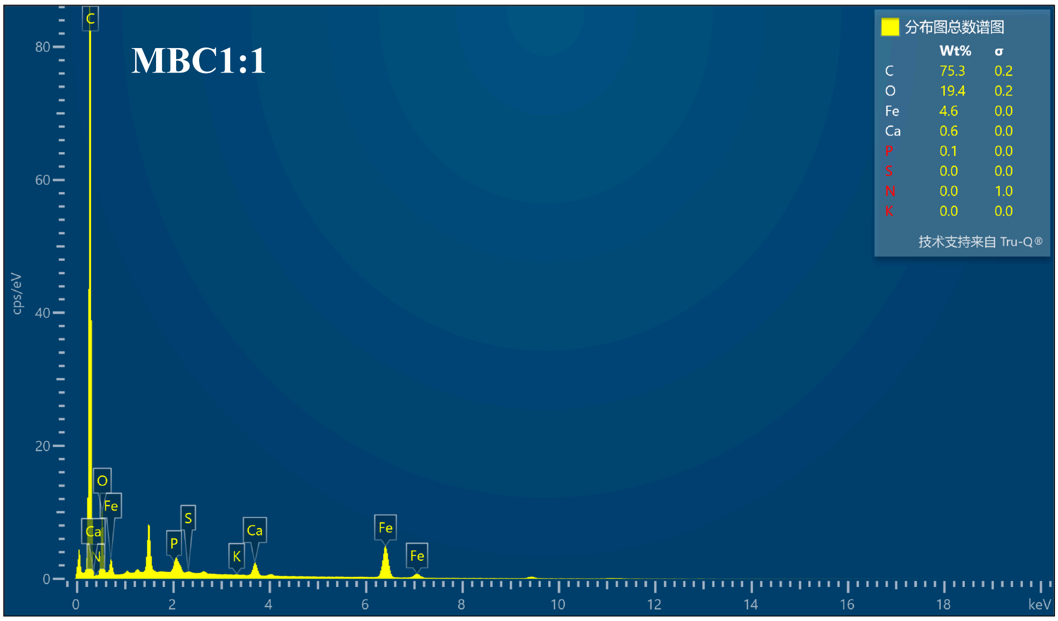
MBC1:1
分布图总数谱图
Wt% σ
C 75.3 0.2
O 19.4 0.2
Fe 4.6 0.0
Ca 0.6 0.0
P 0.1 0.0
S 0.0 0.0
N 0.0 1.0
K 0.0 0.0
技术支持来自 Tru-Q®
cps/eV
keV

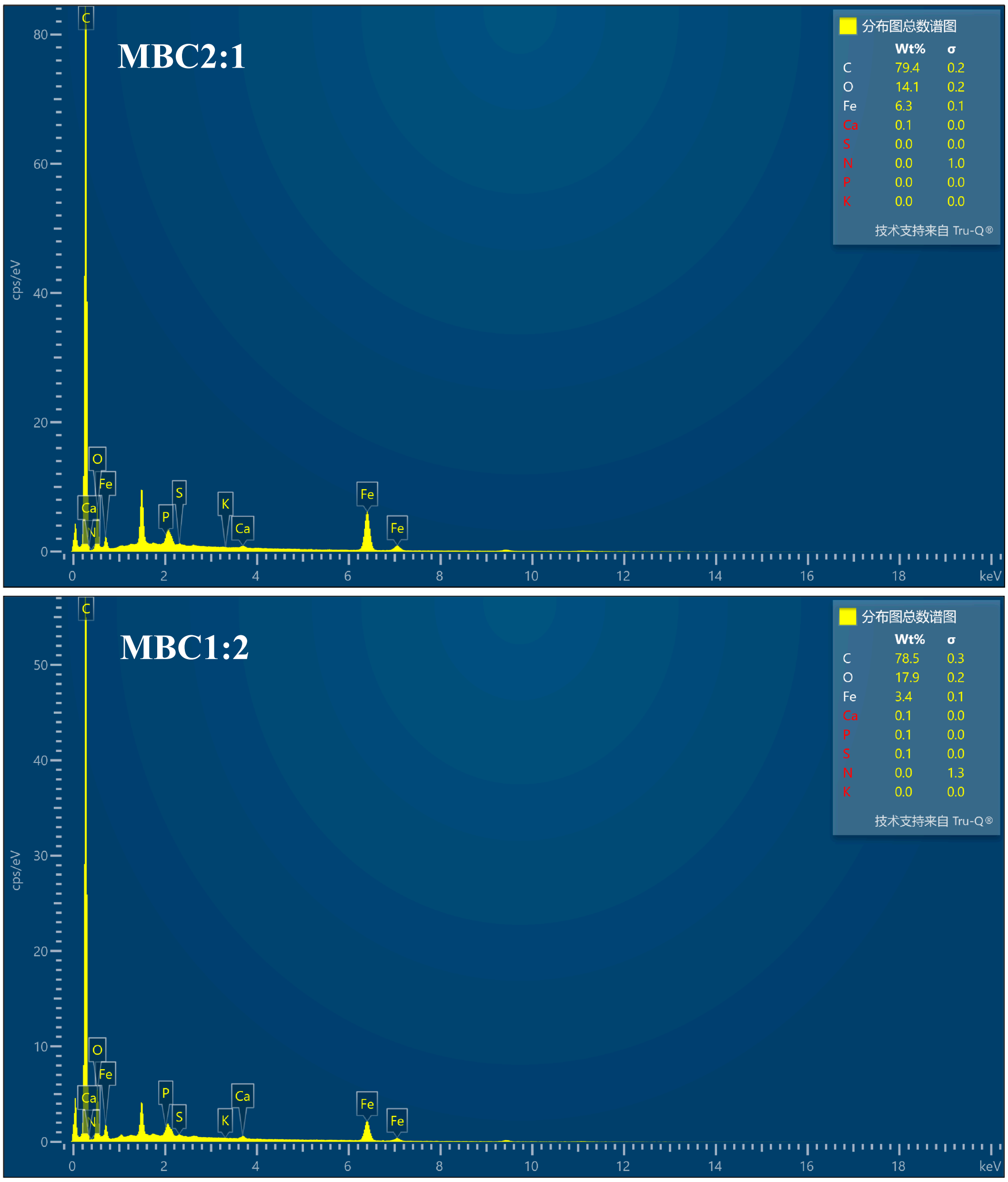

Figure S1. **EDS image of BC, MBC1:1, MBC2:1, and MBC1:2.**

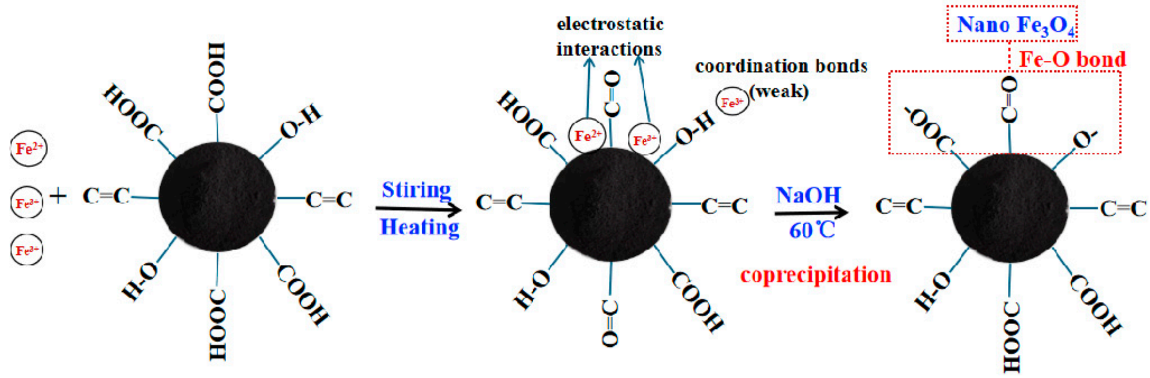

Figure S2. The mechanism of magnetic formation.

Table S1 Experimental data at equilibrium

Mass (mg)	c_0 (mg/L)	Adsorption time(min)	c_e (mg/L)	q_e (mg/g)
120	30	120	0.56	49.39
	40		1.54	65.228
	50		3.52	77.78
	70		10.68	100.202
	90		17.47	122.018
	110		24.01	143.629
	130		34.88	158.022

120 mg of MBC1:1 were added to 200 mL of CR dye solution at 30-200 mg/L. Gently stir the mixture solutions under stirring conditions at ambient temperature. The concentration of CR dye in samples that were taken at regular intervals of every 10 minutes were messured using an ultraviolet spectrometer (UV-5500PC) at the peak absorption wavelength of 498 nm.

Table S2 Experimental data of Recyclability and regeneration of MBC1:1 adsorbent

Numbers of cycle	removal rate (%) of Multiple experimental repetitions						average value of removal rate (%)	SD
	1	2	3	4	5	6		
1	91.63	92.18	92.73	93.28	93.83	94.38	92.73	1.1
2	84.65	85.3	85.95	86.6	87.25	87.9	85.95	1.3
3	69.15	69.75	70.35	70.95	71.55	72.15	70.35	1.2
4	68.71	69.26	69.81	70.36	70.91	71.46	69.81	1.1

A one-way Analysis of Variance (ANOVA) was conducted to investigate the effect of numbers of cycle on removal efficiency. Results indicatedastatistically significant differenceinscores acrossmusic types, $F(3,21)=1751.40$, $p<0.001$. There is a significant difference between the first cycle ($R=92.73$, $SD=1.1$) and each of the second ($R=85.95$, $SD=1.3$), third ($R=70.35$, $SD=1.2$), and fourth ($R=69.81$, $SD=1.1$) cycles. Additionally, significant differences are observed between the second and third cycles, as well as between the third and fourth cycles. However, there was no significant difference the third and fourth cycles ($p=0.69$). It is evident from the results that the removal rate decreased significantly during the first three cycles, but remained relatively stable thereafter.
